# The 1-2-3 cm Advancement Flap Rule in Scalp Reconstruction

**DOI:** 10.7759/cureus.35301

**Published:** 2023-02-22

**Authors:** Hani Shash, Sawsan Marzouq, Abdulrahman Alghamdi, Majd Alrayes, Saud K Alkhaldi, Hayam Shash, Ahmed M Abu Quren

**Affiliations:** 1 Plastic Surgery, King Fahad University Hospital, Al Khobar, SAU; 2 Medicine, Imam Abdulrahman Bin Faisal University, Dammam, SAU

**Keywords:** plastic surgery, tissue expansion, galea score, scalp defects, scalp reconstruction

## Abstract

Background

Defects of the scalp are surgically challenging for several reasons: anatomical convexity limits tissue displacement, resistance to advancement is different at different points on the scalp, and there is also interindividual variation. For many patients, the idea of undergoing an advanced surgery such as a free flap is not preferred. Hence, a simple technique with a favorable outcome is needed. We hereby introduce our new technique: the 1-2-3 scalp advancement rule.

Objectives

The objective of this study is to discover a novel way to reconstruct scalp defects secondary to trauma or cancer, without having the patient undergo a big procedure.

Material and Methods

A total of nine cadaveric heads were used to test the idea of achieving greater advancement and increased scalp mobility to cover a 4×8 cm-sized defect using our proposed 1-2-3 scalp rule. Three steps performed were advancement flap, galeal scoring, and removal of the outer table of the skull. The measurement of advancement was recorded after each step, and the results were analyzed.

Results

The mobility of the scalp was calculated from the sagittal midline with identical arcs of rotation. With zero tension, we found that the total distance of advancement with a flap had a mean of 9.78 mm, while the advancement for the same flap after galeal scoring had a mean of 20.5 mm, and after removing the outer table, the mean advancement was 30.2 mm.

Conclusion

To create a tension-free closure necessary for optimal outcome for scalp defects, our study showed that increased distances were possible using galeal scoring and outer table removal, increasing the distance of advancement by 10.63 mm and 20.42 mm, respectively.

## Introduction

Scalp defects can be attributed to a variety of etiologies, such as excision of benign or malignant tumors, infections, or even radiation necrosis, which are more commonly seen among the older age groups. While in younger patients, trauma and burns are more common [1].

The reconstruction of scalp defects can be challenging for several reasons: limited tissue displacement and variable degrees of resistance at different points on the scalp, as well as individual factors such as age, sex, and comorbidities [2]. In order for a surgeon to perform an optimal reconstruction, the reconstructive plan should take into account the patient’s own preferences and intrinsic and extrinsic factors including age, smoking, the use of corticosteroids, previous surgical incisions, cerebrospinal fluid (CSF) leakage, defect size and location, local tissue quality, hair distortion, radiation, the presence or absence of periosteum, the location of the hairline, and the presence of other comorbidities [3-5].

Primary closure can be performed in scalp defects as long as the defect is less than 3 cm in size and the defect area is loose. Skin grafts can also be done to reconstruct scalp injuries even if the defect is large, provided that the pericranium is intact. In addition, local flaps can also be done to reconstruct and restore shape to a scalp [5]. Transposition, rotation, and advancement flaps are the three types of local flaps. Flaps are usually done in patients who have not been exposed to radiation in the scalp area and cannot undergo more simple procedures such as primary closure. Local flaps are generally safe and have complication rates as low as 3.4% [6]. Regional flaps have limited usage in scalp reconstruction, except for the temporoparietal fascial flap. Regional flaps can be used in patients who need a large amount of vascularized tissue but cannot undergo free tissue transfer. Free tissue transfer is the most commonly used form of reconstruction in total and near-total scalp defects. Free flaps are also used in cases where the scalp has been irradiated, exposure of the dura, a history of chronic infection, or usage of alloplastic material [7]. The main disadvantage of free tissue transfer is aesthetics, due to alopecia and mismatch in both color and contour [8].

The complications of scalp reconstruction are common and might have devastating results in any surgical method and should always be considered. Yet, it could possibly be prevented in many ways, ranging from simple primary prevention to changing the whole surgical approach. However, scalp reconstruction complications may include calvarial desiccation, sequestration, sepsis, extradural hematoma, and swelling due to liquorrhea [7-9]. Important presumptions should be considered for a successful management of scalp defects and to prevent such complications, which include durable coverage, preservation of blood supply, adequate debridement, and proper wound debridement [8,10]. Local scalp flaps, skin grafts, and free tissue transfer remain the preferred reconstruction method in most cases [8]. In patients with aggressive scalp malignant neoplasm, more basic reconstructions, such as skin grafting, may be best used [11]. On the other hand, in patients with deeper wounds with calvarial destruction, custom-fabricated alloplastic cranioplasty prostheses, hard-tissue replacement materials, bone grafts, resorbable materials, or titanium plates are frequently used during reconstructions [12].

Even with this variety of reconstructive methods, scalp defect reconstruction remains a big challenge in some cases. Hence, a simple technique with favorable outcome is needed. We hereby introduce our new technique: 1-2-3 scalp advancement rule.

## Materials and methods

A total of nine cadaveric heads were utilized to test the idea of achieving greater advancement and increased scalp mobility to cover a 4×8 cm-sized defect using our new proposed 1-2-3 scalp advancement rule. Three steps were performed to reach our expected goal: advancement flap, galeal scoring, and removing the outer table of the skull. The advancement was measured after each step, and the results were analyzed and discussed. All of the nine cadaveric heads underwent the same technique of dissection as outlined. A horizontal line connecting the root of the right and left helices was drawn. A second vertical line bisecting the horizontal line was drawn. Then, a 4×8 cm-sized defect was created. A posteriorly based flap of size 8×14 cm^2^ was raised. A unipedicled flap was advanced anteriorly, and the advancement was measured. Then, the galea was scored. Finally, the outer table of the skull was removed, and the total advancement was measured.

Statistical analyses

Statistical analysis was carried out with the Statistical Package for Social Sciences (SPSS) (IBM SPSS Statistics, Armonk, NY) version 23.0. The data were extracted from nine cadaveric heads and were analyzed to find out whether galeal scoring and the removal of the outer table will cause a significant increase in advancement. Descriptive statistics enable you to summarize complex data sets in very few words and using only very basic and easy-to-understand concepts. Two sample paired t-tests were used to measure the difference of the means of the variables and how they differ significantly. All statistical tests that were performed considered statistical significance at P-values<0.05.

Ethical approval

The ethics committee of Imam Abdulrahman Bin Faisal University’s institutional review board (IRB) approved this study (IRB-2021-01-213).

## Results

In Table 1, the advancement flap was measured in millimeters after galeal scoring and the “removal of the outer table with galeal scoring.” The defect size was 8×4 cm^2^ and the flap 8×14 cm^2^. The mobility of the scalp was calculated from the sagittal midline with identical arcs of rotation. With zero tension, we found that the total increase in advancement with a flap had a mean of 9.78 mm, while the advancement for the same flap after the galeal score had a mean of 20.56 mm. After removing the outer table, we observed a mean advancement of 30.22 mm, reporting that the largest advancement seen was after removing the outer table with galeal scoring with a confidence interval (CI) of 29.36-31.08 mm (5.5%).

**Table 1:** Measured advancement flap in millimeters to scalp defect after “galeal scoring” or the “removal of the outer table/galeal scoring”

Case	Defect (cm^2^)	Flap (cm^2^)	Advancement flap (mm)	Advancement flap after galeal scoring (mm)	Advancement flap after the removal of the outer table with galeal scoring (mm)
1	8×4	8×14	9	20	30
2	8×4	8×14	10	21	31
3	8×4	8×14	11	19	29
4	8×4	8×14	9	20	32
5	8×4	8×14	10	21	28
6	8×4	8×14	11	22	29
7	8×4	8×14	9	23	30
8	8×4	8×14	9	20	31
9	8×4	8×14	10	19	32
Mean			9.78	20.56	30.22

Table 2 is the comparison between the advancement flaps before and after galeal scoring. As shown in Table 2, there is a significant advancement in comparing the mean between advancement flap before and after galeal scoring. The P-value was less than 0.05 with confidence interval (CI) of 9.516-12.040.

**Table 2:** Comparison of mean between advancement flap after galeal scoring and advancement flap only AF, advancement flap; GS, galeal scoring; df, degree of freedom

Paired sample test									
		Paired differences					T	df	Significance (two-tailed test)
		Mean	Standard deviation	Standard error mean	95% confidence interval of the difference				
					Lower	Upper			
Pair 1	AF with GS-AF	10.778	1.641	0.547	9.516	12.040	19.698	8	0.0000000459

In addition to the galeal scoring, the removal of the outer table added an extra 10 mm when performed correctly. When we compare the mean between advancement flap after the removal of outer the table and galeal scoring with advancement flap only (Table 3), it shows a significant result with P-value less than 0.05 and CI of 18.950-21.938, which means that the advancement flap after the removal of the outer table with galeal score is greater than advancement flap only.

**Table 3:** Comparison of mean between advancement flap after the removal of the outer table and galeal scoring and advancement flap only AF, advancement flap; OT, outer table; GS, galeal scoring; df, degree of freedom

Paired sample test									
		Paired differences					T	df	Significance (two-tailed test)
		Mean	Standard deviation	Standard error mean	95% confidence interval of the difference				
					Lower	Upper			
Pair 1	AF after OT and GS-AF with GS	0.444	1.944	0.648	18.950	21.938	31.556	8	0.0000000011

Table 4 shows the comparison between the mean advancement flap after the removal of the outer table and galeal scoring with advancement flap with galeal scoring. The P-value is less than 0.05, and CI is 7.948-11.385, which indicate a significant result, and thus, the advancement flap after the removal of the outer table with galeal score is greater than the advancement achieved with galeal score. Thus, when both techniques were combined, we ended up with a greater advancement flap.

**Table 4:** Comparison of mean between advancement flap after the removal of the outer table and galeal scoring and advancement flap with galeal scoring AF, advancement flap; OT, outer table; GS, galeal scoring; df, degree of freedom

Paired sample test									
		Paired differences					T	df	Significance (two-tailed test)
		Mean	Standard deviation	Standard error mean	95% confidence interval of the difference				
					Lower	Upper			
Pair 1	AF after OT and GS-AF with GS	9.667	2.236	0.745	7.948	11.385	12.969	8	0.0000011837

**Figure 1:**
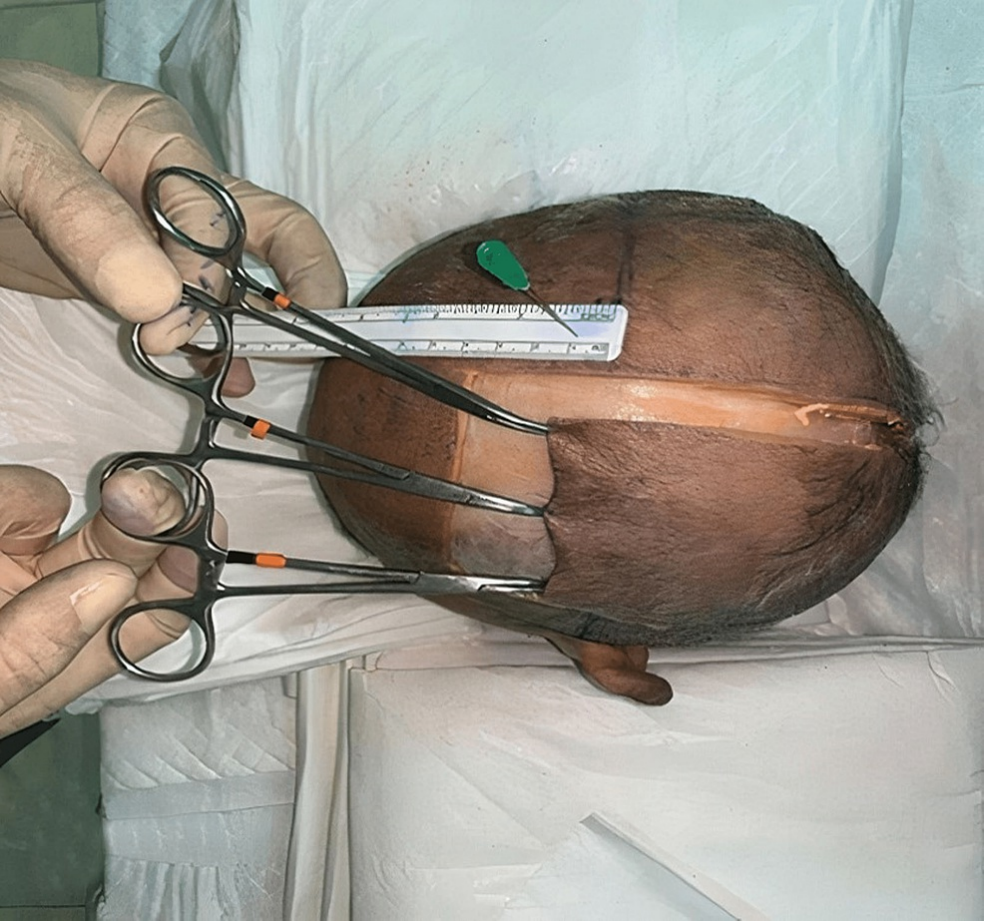
Advancement made without galeal scoring or burring of the outer table

**Figure 2:**
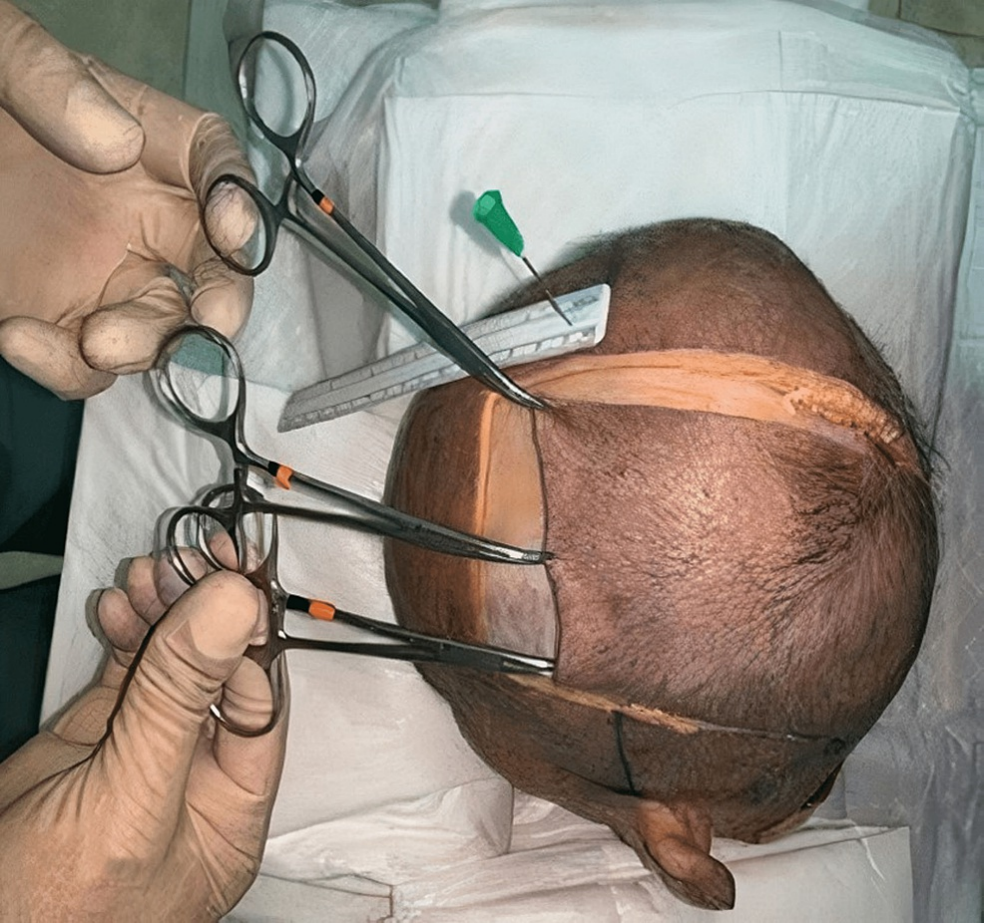
Advancement made with galeal scoring

**Figure 3:**
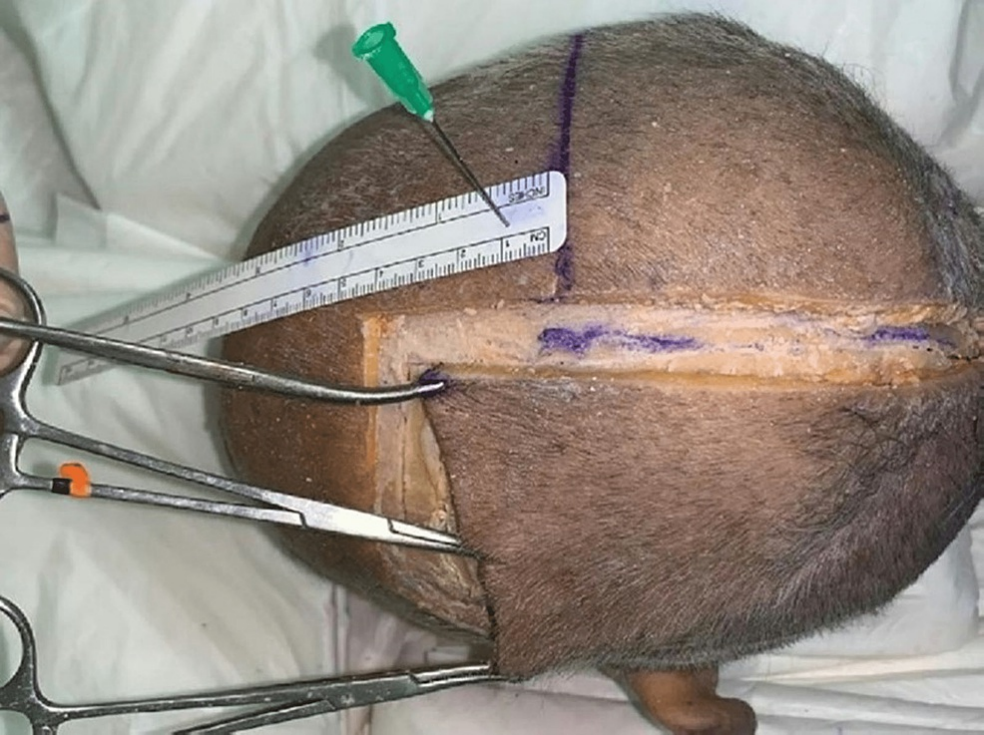
Advancement of 3 cm made after the removal of the outer table

**Figure 4:**
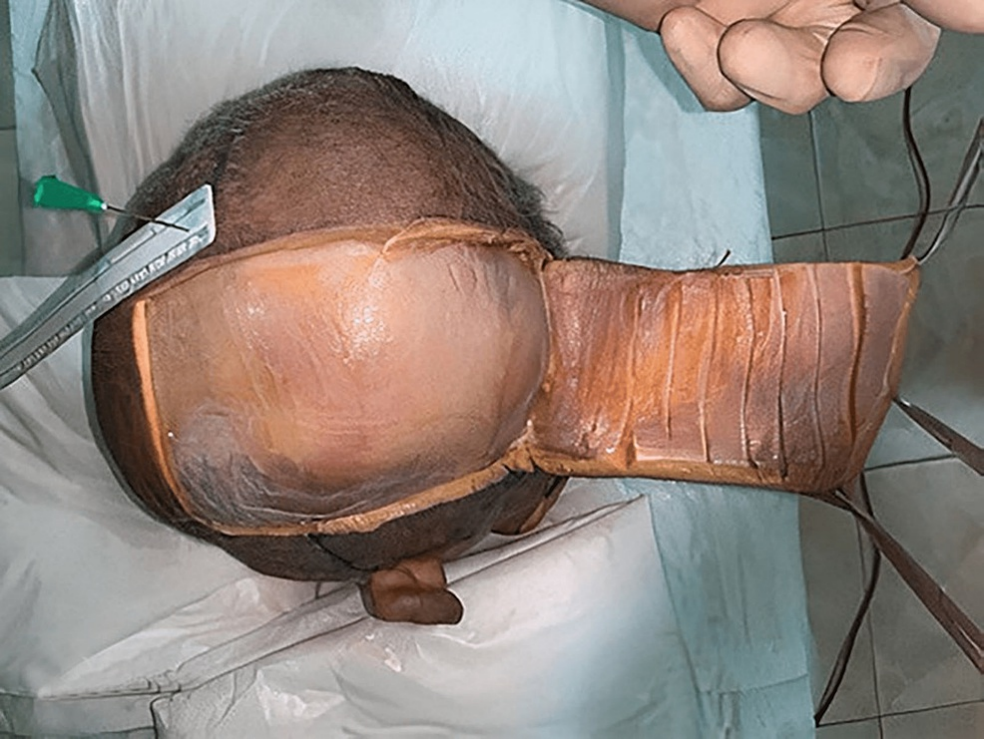
Incisions of the galea at 1 cm intervals

**Figure 5:**
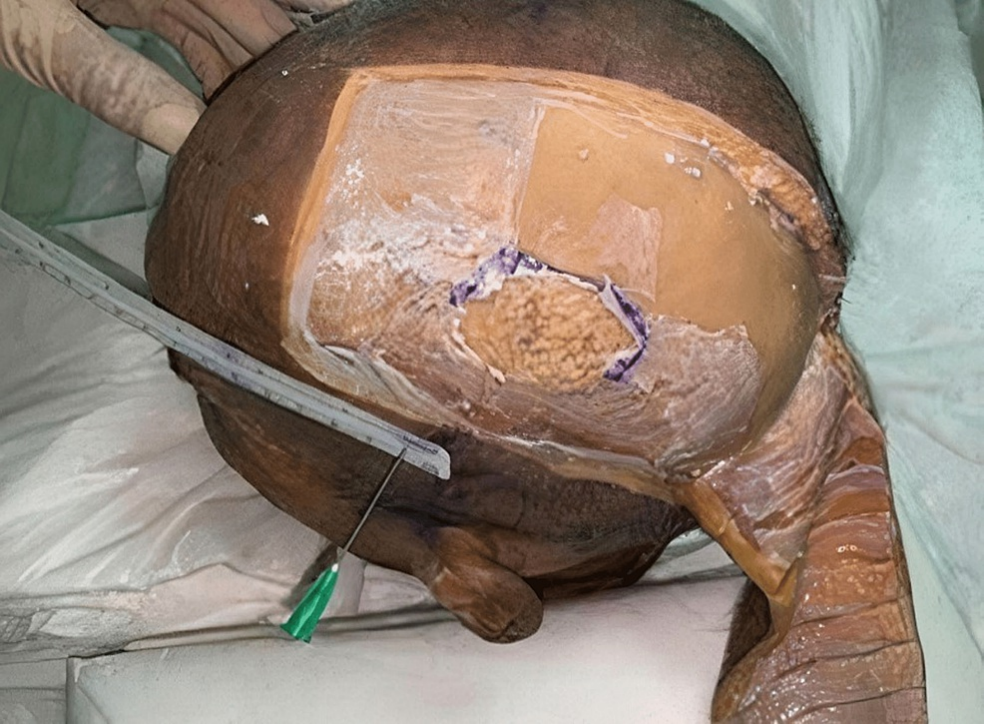
Removal of the outer table to further increase the advancement flaps

The following figures show the experiment of our study. In Figure 1, advancement flap was made without galeal scoring or the removal of the outer table. In contrast, in Figure 2, advancement flap was made with galeal scoring. Figure 3 shows advancement of 3 cm made after burring of the outer table. Figure 4 shows incisions of the galea at 1 cm intervals. Figure 5 shows the removal of the outer table to further increase the advancement flaps.

## Discussion

Primary closure is the preferred therapeutic strategy for small scalp defects in scalp restoration, followed by secondary healing. However, due to the limited elasticity of scalp tissues, only less than 3 cm in length is usually amenable to direct closure [12]. Tissue expansion is another approach for scalp repair. Using this procedure, scalp abnormalities can be repaired with hair-bearing skin with minimum cosmetic disfigurement. Unfortunately, quick skin expansion is rarely achievable in oncologic patients. Furthermore, the adoption of this approach is associated with a significant complication risk in patients with a history of previous radiation, infection, or alloplastic reconstruction [13].

Several free flaps have been described in the previous decades to reconstruct the scalp. In this context, the key criteria that influence flap type selection are defect size, recipient vessel, and pedicle length [14]. The latissimus dorsi flap is usually referred to as the first choice in big scalp defect reconstruction [15,16]. The advancement flap, in which the skin segment advances directly to the area that needs to be repaired without rotation or translation movement, is another form of flap that is also particularly efficient in scalp restoration [6]. The omental free flap, which is covered in a skin graft, has been used for both subtotal and total scalp restoration. The best reconstruction method for really large scalp defects is occasionally free flaps [15].

According to one study, a scalp free flap can survive radiation therapy following surgery for the removal and repair of a malignant tumor [16]. In our study, we proposed our new method of scalp defect reconstruction, “1-2-3 rule,” in which we attempted to close an 8×4 cm^2^ scalp defect. Usually, defects more than 3 cm^2^ carry a significant challenge [15,17], yet as we have demonstrated with our proposed method, the results were promising. The “1-2-3 rule” consists of three consecutive steps, in which each adds 1 cm to the advancement of the flap, with a total of 3 cm when all combined. Galeal scoring is a technique that we used in addition to the removal of the outer table of the skull. It has been reported that each galeal scoring of 1 cm long will improve the flap length by 1.67 mm along with a 40% decrease in wound tension [15,17-20]. Our proposed 1-2-3 scalp advancement rule is different from the 1-2-3 rule proposed by Russo in his study, which was done on patients with defects ranging from 1 to 4 cm where defects that had a distance between 1 and 2 cm required a single relaxation incision, where two relaxation incisions were required for defects between 2 and 3 cm and three incisions for defects between 3 and 4 cm [6]. The results of our study are similar to a study conducted by Ibrahim et al. in 2016, except that our study had nine cadaveric heads compared to two heads used in their study [4].

Scalp reconstruction includes differences according to regions. The parietal region was selected in the study, and it is the area where simple reconstruction methods are most frequently used [18]. For example, an 8×4 cm defect in the parietal region can be closed with a smaller rotation flap instead of an 8×14 cm advancement flap because the parietal region is the loosest area of the scalp. Although the gain obtained in the third preferred method is higher than the other methods, its clinical applicability is limited. Because scraping of the outer tabula increases the risk of bleeding and may cause hematoma and flap loss, it may also result in intracranial penetration in inexperienced hands [16].

Limitation and recommendation

In our study, we used cadavers, which of course have different elasticity in comparison to the viable skin of the living body. Therefore, our findings might be different when applied on living patients. We believe that our proposed method has demonstrated reproducible and satisfactory results with possible favorable outcomes when applied on real patients. Thus, we recommend further studies to be performed on alive patients in order to develop a new approach that can be added to the reconstructive ladder in reconstructive surgeries involving the scalp.

## Conclusions

For patients with scalp defects, tension-free scalp closure is necessary to achieve the best possible outcome. In this study, we showed that the 1-2-3 rule of scalp advancement made galeal scoring and removing the outer table sufficient enough to cover the defect and therefore does not necessitate performing complex surgeries such as a free flap to cover scalp 4×8 cm defects.
